# Alternative splicing of apoptosis-related genes in imatinib-treated K562 cells identified by exon array analysis

**DOI:** 10.3892/ijmm.2011.872

**Published:** 2011-12-29

**Authors:** JING LIU, YUN XIAO, HUO-MEI XIONG, JING LI, BO HUANG, HAI-BIN ZHANG, DAN-QIN FENG, XI-MIN CHEN, XIAO-ZHONG WANG

**Affiliations:** 1 Department of Clinical Laboratory, Second Affiliated Hospital of Nanchang University, Nanchang 330006; 2 Department of Clinical Laboratory, First Affiliated Hospital of Nanchang University, Nanchang 330006, P.R. China

**Keywords:** alternative splicing, exon splicing, apoptosis, expression profiling, microarray

## Abstract

Imatinib is the therapeutic standard for newly diagnosed patients with chronic myeloid leukemia (CML). In these patients, imatinib has been shown to induce an apoptotic response specifically in cells expressing the oncogenic fusion protein BCR-ABL. Previous studies in our lab revealed that imatinib-induced apoptosis in K562 cells involves a shift in production of Bcl-x splice isoforms towards the pro-apoptotic Bcl-x_S_ splice variant. Here, we report the findings from our subsequent study to identify other apoptosis-related genes that are differentially spliced in response to imatinib treatment. Gene expression profiling of imatinib-treated K562 cells was performed by the Affymetrix GeneChip^®^ Human Exon 1.0 ST array, and differences in exon-level expression and alternative splicing were analyzed using the easyExon software. Detailed analysis by reverse transcription-PCR (RT-PCR) and sequencing of key genes confirmed the experimental results of the exon array. Our results suggest that imatinib treatment of K562 cells causes a transcriptional shift towards alternative splicing in a large number of apoptotic genes. The present study provides insight into the molecular character of apoptotic leukemia cells and may help to improve the mechanism of imatinib therapy in patients with CML.

## Introduction

Chronic myeloid leukemia (CML) is a hematopoietic stem cell disorder characterized by marked increase in granulocytes, bone marrow hyperplasia, and splenomegaly. The current annual incidence of CML has been reported to be 1 to 2 cases per 100,000 people, and accounts for 15% of adult leukemias diagnosed worldwide (1). The molecular hallmark of CML is the Philadelphia (Ph) chromosome, a reciprocal translocation t (9:22) (q34; q11) between the proto-oncogene ABL on chromosome 9 and the BCR gene on chromosome 22. This abnormality has been clinically detected in 95% of CML patients, and is considered the primary risk factor for CML development (2). The Ph translocation creates a fusion *BCR-ABL* oncogene, which encodes a functional protein with constitutive tyrosine kinase activity that aberrantly modulates tumorigenic pathways.

Imatinib is the first chemotherapeutic agent described as a specific BCR-ABL tyrosine kinase inhibitor. Its proven efficacy for the treatment of patients with CML has led to its preferred use as first-line treatment, supplanting the traditional single agents busulphan, hydroxyurea and interferon-α (3). The molecular mechanism of imatinib has been described: imatinib potently binds to the inactive form of BCR-ABL, thereby blocking the ATP-binding site and its enzymatic activity (4). Imatinib treatment of CML patients has produced estimated cumulative best rates of complete hematologic response (CHR) and complete cytogenetic response (CCyR) of 98 and 87%, respectively (5). Despite the significant improvements in the outcome for patients with CML following treatment with imatinib, the exact mechanism of its anti-leukemic effect remains to be completely elucidated (6).

In a previous study, we discovered that imatinib treatment of K562 leukemia cells produced a significant change in the ratio of Bcl-x splice variants. The Bcl-x gene undergoes alternative splicing at exon 2 to produce two isoforms with distinctive anti- and pro-apoptotic properties. The Bcl-x_S_ isoform promotes apoptosis, while the Bcl-x_L_ inhibits apoptosis. Imatinib treatment of K562 cells led to a transcriptional shift towards the Bcl-x_S_ isoform, which in turn promoted apoptosis in the treated cells. Accordingly, this finding suggested that imatinib could regulate the alternative splicing of other apoptotic genes in K562 cells.

Alternative splicing is a common mechanism used by the human system to generate diversity in the transcriptome and proteome. Through alternative splicing, multiple different transcripts can be generated from a single mRNA precursor. Recent studies have suggested that up to 94% of all human genes undergo alternative splicing, and aberrant pre-mRNA splicing has been implicated in the pathogenesis of neoplasia (7,8). Likewise, studies have demonstrated that most of the currently used chemotherapeutic agents not only induce an apoptotic response, but also affect the alternative splicing of some genes. Shkreta *et al* (9) determined that 20 of the mainstream anticancer drugs could influence the production of Bcl-x splice isoforms. In addition, these drugs could alter a subset of alternative splicing events in some cell lines, suggesting that the influence of anticancer drugs on alternative splicing is a very common phenomenon.

In recent years, powerful techniques for genome-wide identification and analysis of alternative splicing isoforms have been developed. These large-scale high-throughput analytical methods have been successfully applied for the identification of differential splicing events in various cancer tissues (10). Exon arrays, which contain both known and predicted exons, are rapidly gaining popularity and becoming a standard for both gene- and exon-level expression analyses (11). The Affymetrix GeneChip^®^ Human Exon 1.0 ST array (Human Exon 1.0 ST array) is the latest product in the family of exon arrays. It contains approximately 5.4 million probes grouped into 1.4 million probe sets, allowing the interrogation of over 1 million exon clusters, which are exon annotations from various sources that overlap by genomic location. For each gene, the average number of probes on the exon array is 35, and these probes are usually distributed along the entire transcript sequence. The commercial availability of specific software for exon-level expression analysis has provided a powerful tool for the study of alternative splicing for every known and predicted exon. At the same time, exon arrays provide robust gene-level expression analysis (Affymetrix Technical notes, Identifying and Validating Alternative Splicing Events) facilitating identification of relatively subtle differences in the isoform expression ratios.

In our current study, we employed the Human Exon 1.0 ST Array to investigate the comprehensive profile of apoptotic genes in leukemia K562 cells that are differentially spliced in response to imatinib treatment.

## Materials and methods

### Cell culture

Human myeloid leukemia cells K562 were grown in Roswell Park Memorial Institute 1640 (RPMI-1640; Hyclone, USA) media supplemented with 10% fetal bovine serum (FBS; Hyclone) in a 5% CO_2_ humidified atmosphere at 37°C. Experimental cultures were initiated at a density of 2×10^5^ cells/ml. Imatinib (Novartis Co., China) was added from a 1 mM stock solution in DMSO to achieve various final concentrations in culture. The control non-treated cells were generated by adding an equal volume of DMSO alone. Cells were incubated for up to 24 h before cell proliferation was assessed by WST-1 reagent based assay kit (Millipore, USA) and apoptosis was assessed by flow cytometry. For details of the two methods, refer to our previous study (12).

### RNA extraction and array hybridization

Total-RNA was extracted from 10^7^ K562 cells by using the TRIzol reagent (Tiangen, China) following the manufacturer’s instructions. The extracted RNA was further purified by DNase I treatment using an RNeasy mini kit and an RNase-Free DNase Set (Qiagen, China), according to the manufacturer’s protocols. The concentration and purity of total-RNA were determined by spectrophotometric measurements at an optical density (OD) of 260/280 nm and electrophoresis on 1% agarose gel. An OD_260_/OD_280_ ratio of 1.8–2.0 was considered to indicate pure RNA. RNA samples were immediately frozen and stored at −80°C until required for use in further microarray studies.

Two 1-μg RNA samples from untreated control K562 cells and imatinib-treated K562 cells (1 μM of imatinib for 24 h) were labeled according to the GeneChip whole transcript (WT) Sense Target Labeling Assay as provided by the manufacturer (http://www.affymetrix.com). Labeled RNA was hybridized to the Human Exon 1.0 ST Arrays for 16 h, following the Affymetrix protocol. Afterwards, arrays were scanned using the Affymetrix GCS 3000 7G Scanner and digital images were processed by the GeneChip^®^ Operating Software v1.4 to produce raw CEL intensity files for analysis of differential expression.

### Data analysis

Data from the exon arrays were processed using the easyExon software (http://microarray.ym.edu.tw/easyexon). This tool provides a user-friendly, platform-independent and efficient processing method to explore Affymetrix exon array data. Two parallel analyses (gene-level and exon-level) were carried out by this software. To improve the processing efficiency, we downloaded the related human gene database and meta probeset files from the software homepage (http://microarray.ym.edu.tw:8080/easyexon/index.jsp?mode=support) and installed each as a local copy.

After data loading of the CEL files, gene/exon signal summarizations were calculated automatically by the Affymetrix Power Tools (APT) package, which can execute the binary file ‘apt-probeset-summarize’ to generate both exon-level and gene-level summary files. Two of the most commonly used signal estimation algorithms, RMA and PLIER, were implemented to combine information from probes belonging to the same transcript, or exon, to generate a summarized expression signal value of the single represented gene or exon (13). Background noise was detected using the ‘Detection above Background (DABG)’ algorithm. Normalization was performed by the ‘quantile normalization’ algorithm for both the exon- and gene-level.

### Reverse transcription-PCR (RT-PCR) and sequencing validation

RT-PCR and direct sequencing were used to confirm the differentially expressed alternatively spliced genes detected by the Human Exon 1.0 ST Arrays. Reverse transcription was performed with 1 μg of total-RNA using a reverse transcription kit (Takara Bio, Inc., Japan), following the manufacturer’s instructions. For subsequent PCR analysis, 1 μl of cDNA was used. The primers and reaction conditions for each transcript are shown in Table I. The amplified products were electrophoresed through 1.5–2.5% agarose gels, according to amplified fragment size requirements for resolution. Images of the gels were analyzed by the Quantity One 4.6.2 software (Bio-Rad, USA). Ratios between splice variants expressed in untreated and imatinib-treated cells were calculated. Distinct gel bands were purified with a gel extraction kit (Tiangen), sequenced using the same primers as for PCR and the BigDye Terminator v3.1 Cycle Sequencing kit (Applied Biosystems, USA), and analyzed on an ABI PRISM 3100 Genetic Analyzer (Applied Biosystems).

## Results

### Exon-level expression differences induced by imatinib treatment

The pro-apoptotic activity of imatinib on K562 cells was first confirmed by a WST-1 assay and a double fluorescence assay to analyze cell proliferation and apoptosis, respectively. Our results revealed that imatinib exposure rapidly induced proliferation inhibition and apoptosis of K562 cells in a time-dependent manner [data not shown; identical to that of our previous study (12)].

The expression of alternatively spliced genes in the K562 cells in response to treatment with imatinib were then assessed via exon array and easyExon analysis. Before performing statistical filtration, the limitation number of probesets in a transcript cluster was set from 4 to 40. For any transcript cluster with probesets greater than the setting number, only the setting number of probesets was included in the latter analysis. Three statistical methods for exon-level filtration in the easyExon software were available: Affymetrix MIDAS (Microarray Detection of Alternative Splicing), Partek AS ANOVA (Alternative Splice Analysis of Variance), and PAC (Pattern-Based Correlation) for exon analysis. We used the default Affymetrix MIDAS. For selecting alternatively spliced genes, the criteria for probeset filtration were set as follows, fold-change (FC) ≥2 or ≤0.5, with a statistical significance of P<0.05.

A total of 331 genes represented by one or several probesets met the above criteria. Most of these probesets presented change in expression of less than 3-fold. The genes affiliated with these probesets were investigated by the easyExon transcript cluster filtration tool to determine which were related to cell apoptosis. Briefly, the easyExon ‘GO Biological Process’ parameter was set for ‘apoptosis’ related to transcript-affiliated gene name or accession number. The program identified 175 apoptosis-related genes from the original set of 331. Each of these genes contained at least one probeset meeting the above criteria, for a total of 430 probesets. Tables II and III list the 20 most upregulated and most downregulated probesets.

### Detection of alternative splicing events in response to imatinib treatment

**Figure 1:**
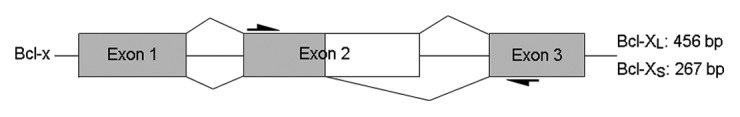

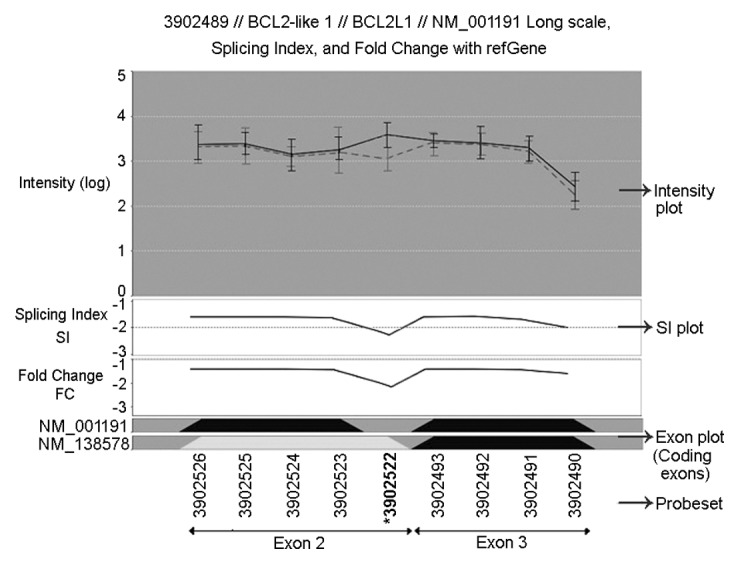

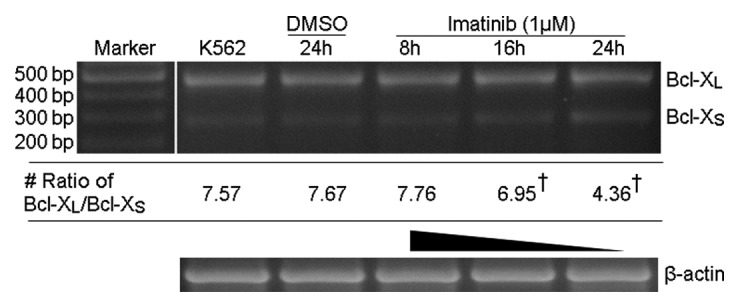
Imatinib regulates Bcl-x splicing in K562 cells. K562 cells were treated with 1 μM of imatinib. Total-RNA was extracted and then RT-PCR was used to validate the alternative splicing of Bcl-x (positive control). (A) Schematic representation of alternative splicing of the Bcl-x exon 2, and the resulting mRNAs. The RT-PCR primer positions are schematically presented (black arrows). (B) The transcript cluster of Bcl-x from the exon array. Transcripts carrying the 3′ end of exon 2 were downregulated in the imatinib-treated K562 cells (black line). This region was targeted by probesets 3902522, and found to be significantly differentially expressed by the MIDAS test (labeled in bold). The fold-change of this probeset between untreated and imatinib-treated cells was greater than 1.5. (C) RT-PCR validation of alternatively spliced Bcl-x. The agarose gel indicates the larger (exon-included) isoforms and smaller (exon-skipped) isoforms. There was a decrease observed in the Bcl-x_L_/Bcl-x_S_ ratio that corresponded with an increase in treatment time. β-actin was used as an internal control. Maker, 100-bp ladder. ^#^Average ratios (n=3); ^†^P<0.01 vs. untreated K562 cells.

EasyExon provides a module to visualize and interpret selected subsets of exons and transcripts that meet specified criteria. The normalized, log-transformed and variance stabilized probeset intensities of a transcript are plotted as signal mean ± standard error in an intensity plot. Probesets marked with asterisks by the program represent statistically significant alternative splicing events which pass the defined MIDAS/Partek AS ANOVA threshold. Probesets colored in bold by the program indicate cases in which the intensity FC between groups was greater than the threshold (by at least 2-fold). The degree of alternative splicing can also be visualized by the splicing index (SI) plot in the same panel. Different probesets for the same exon group together and are visually indicated by the program using a triangle or trapezoid. Adjacent exons are distinguished by different colors. From the graphic presentation of analysis results, we were able to identify whether a specific isoform was up or downregulated in the imatinib-treated cells (Fig. 1B) (13).

After identifying probesets that were differentially spliced in response to imatinib treatment, we randomly chose three internal exons (RNF34, DAPK1 and SNCA) for experimental validation. In addition, we included Bcl-x as the positive control, which was shown to be differentially spliced in K562 cells treated with imatinib in our previous study (12).

**Figure 2:**
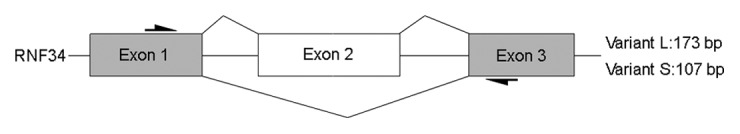

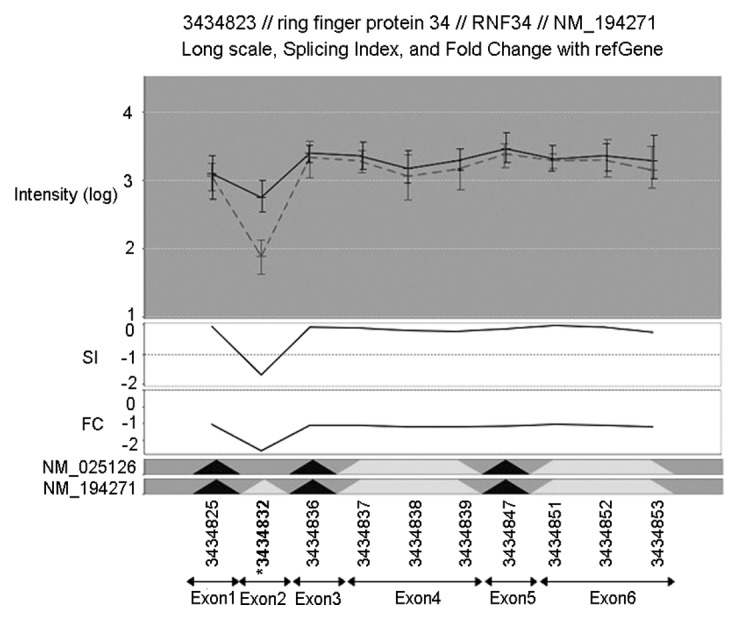

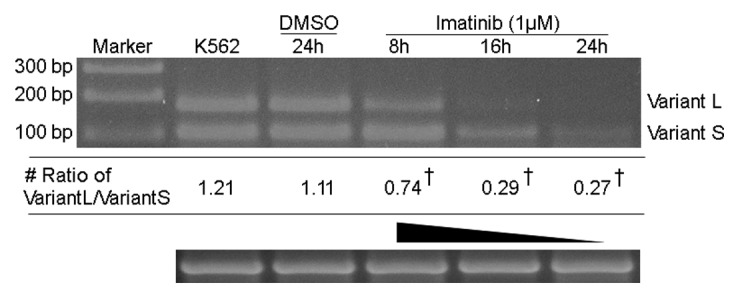

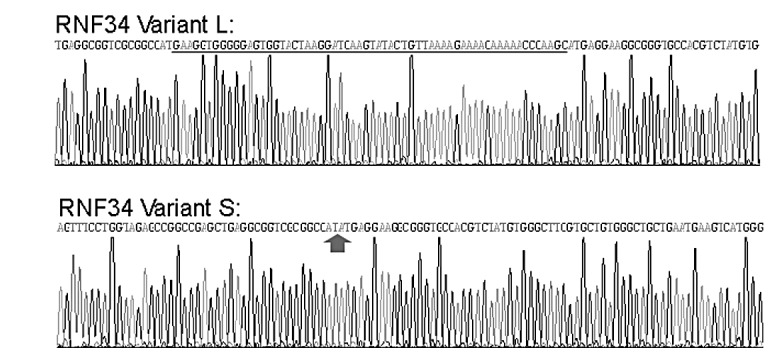
Imatinib regulates RNF34 splicing in K562 cells. K562 cells were treated with 1 μM of imatinib. Total-RNA was extracted and then RT-PCR and sequencing were used to validate the alternative splicing event of RNF34. (A) Schematic representation of alternative splicing of RNF34 exon 2, and the resulting mRNAs. The RT-PCR primer positions are schematically presented (black arrows). (B) The transcript cluster of RNF34. The transcript with exon 2 was downregulated in the imatinib-treated K562 cells (black line). This exon was targeted by probeset 3434832, and was identified as significantly differentially expressed by the MIDAS test (labeled in bold). (C) Representative RT-PCR validation result. Arrows indicate the larger (exon-included) isoforms and smaller (exon-skipped) isoforms. There was a decrease in the variant1/variant2 ratio that occurred along with increasing imatinib treatment time. β-actin was used as an internal control. Maker, 100-bp ladder. (D) Sequencing validation of alternatively spliced RNF34. Bases underlined in black belong to the alternatively spliced exon 2. The gray arrow points to the splicing position. ^#^Average ratios (n=3); ^†^P<0.01 vs. untreated K562 cells. L, long form of RNF34; S, short form of RNF34.

**Figure 3:**
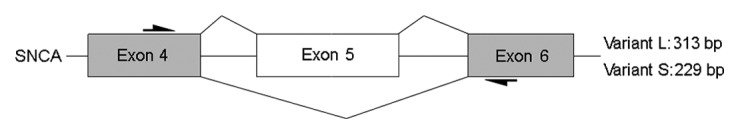

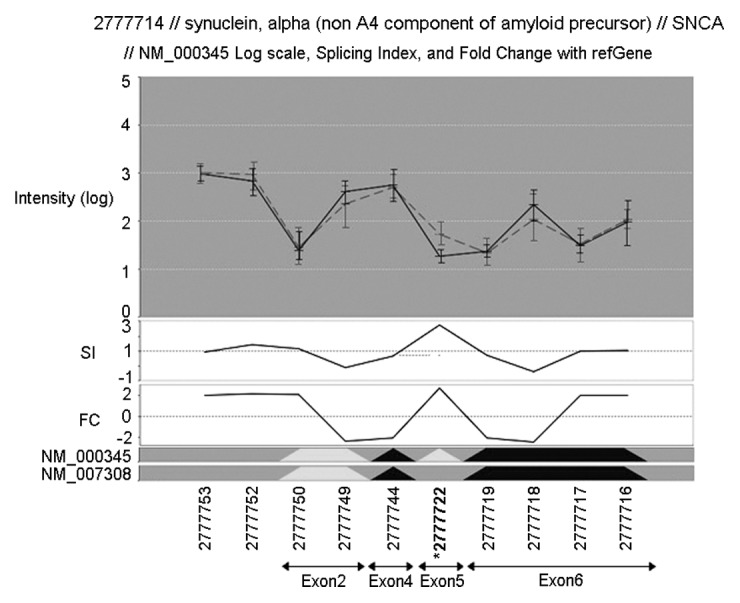

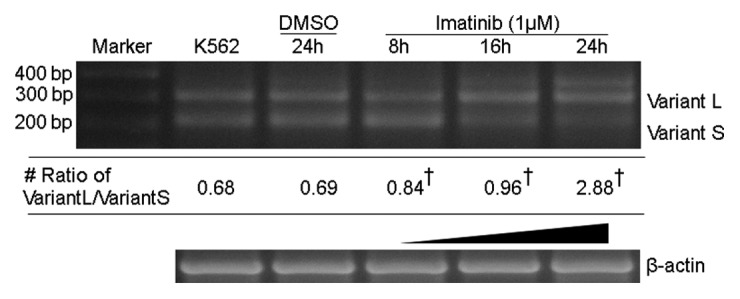

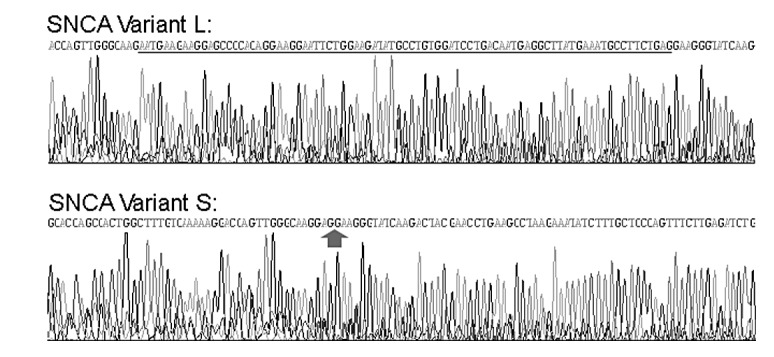
Imatinib regulates SNCA splicing in K562 cells. K562 cells were treated with 1 μM of imatinib. Total-RNA was extracted and then RT-PCR and sequencing were used to validate the alternative splicing of SNCA. (A) Schematic representation of alternative splicing of SNCA exon 5, and the resulting mRNAs. The RT-PCR primer positions are schematically presented (black arrows). (B) The transcript cluster of SNCA. Exon 5 is upregulated in the imatinib-treated K562 cells (black line). This exon was targeted by probeset 2777722 and was identified by the MIDAS test (labeled in bold). (C) Representative RT-PCR validation result. The agarose gel revealed the presence of the larger (exon-included) isoform and smaller (exon-skipped) isoform. There was an increase in the ratio of variant L: variant S along with increase in treatment time. β-actin was used as an internal control. Maker, 100-bp ladder. (D) Sequencing validation of alternatively spliced SNCA. Bases underlined in black belong to the alternatively spliced exon 5. The gray arrow points to the splicing position. ^#^Average ratios (n=3). ^†^P<0.01 vs. untreated K562 cells. L, long form of SNCA; S, short form of SNCA.

We introduce here a workflow for detecting alternative splicing events between untreated K562 cells and imatinib-treated K562 cells by using the easyExon program. In Bcl-x (ID 3902489), the probeset (ID 3902522, in bold) targeting the 3′ region of exon 2 was found to be downregulated in imatinib-treated K562 cells. The expression fold-change of this probeset was larger than 2 (labeled in black) and the difference was statistically significant (marked by an asterisk by the program). This indicated that there were two Bcl-x isoforms in K562 cells, one with both the 5′ and 3′ region of exon 2 and another with only the 5′ region of exon 2, and the latter one was upregulated in imatinib-treated K562 cells (Fig. 1B). In RNF34 (ID 3434823), the second probeset (ID 3434832, in bold) targeting exon 2 was found to be downregulated in imatinib-treated K562 cells. In SNCA (ID 2777714), the probeset (ID 2777722) targeting exon 5 was found to be upregulated in imatinib-treated K562 cells. Both RNF34 and SNCA had two spliceosomes in K562 cells and the alternative splicing was affected by imatinib (Figs. 2B and 3B). Similarly, we were able to identify other transcripts that undergo alternative splicing by using this process.

### RT-PCR and sequencing validation

The expression of Bcl-x, RNF34 and SNCA mRNAs in K562 cells before and after treatment with imatinib were evaluated by RT-PCR using primers targeting the exons that flank the exon of interest. Amplification results were visualized by gel electrophoresis. When two alternatively spliced isoforms were present, two different bands would be produced by the same primer pair between the two groups (Figs. 1A and C, 2A and C, 3A and C). The relative quantity of the transcripts was calculated by the Quantity One software. The results indicated a difference in the expression ratio of the isoforms in response to imatinib treatment for probesets 3902522 (Bcl-x), 3434832 (RNF34) and 2777722 (SNCA). The relative quantity analyses for Bcl-x, RNF34 and SNCA were consistent with the trend of SI values calculated from the exon array data (Figs. 1C, 2C and 3C). To further validate the differences between the spliceosomes, we performed sequencing validation of the distinct bands resolved by gel electrophoresis. The sequencing results for RNF34 and SNCA confirmed the alternative splicing events that were predicted by the exon array data (Figs. 2D and 3D).

For DAPK1, the sequencing validation did not support the presence of alternatively spliced isoforms even though two gel bands that appeared to be in the correct position were detected (data not shown).

## Discussion

Alternative splicing of mRNA precursors is a nearly ubiquitous and extremely flexible point of gene control in humans (14). Abnormal alternative splicing has been shown to be associated with many human cancers (15). In addition, previous studies have demonstrated that some anticancer drugs can affect alternative splicing of genes in cancer cells (9,16). These results may provide a novel rationale for the effectiveness of anticancer drugs.

Apoptosis is a major pathway involved in the complex homeostatic balance between cellular proliferation and cell death. Previous studies have also demonstrated that transcripts from a significant number of genes involved in apoptosis are alternatively spliced, often resulting in isoforms with opposing roles in promoting or preventing cell death (17–19). Examples of this type are found in every category of apoptotic regulators, from transmembrane receptors (Fas, Fas ligand and LARD) and adaptor molecules (Bcl-x, Bak, Apaf1, survivin, Mcl-1 and TRAF2) to caspases (caspase-1, -2, -6, -7, -8 and-9) and executors (FLIP and ICAD) (20). Although alternative splicing is an important mechanism regulating apoptosis, the effect of chemotherapeutic agents on the alternative splicing of apoptotic genes has rarely been examined (9).

In previous studies, we have demonstrated that one of the most commonly used chemotherapeutic agents, imatinib, which is considered the most effective and a relatively safe drug for the treatment of the chronic phase of CML, could regulate alternative splicing of the apoptotic gene Bcl-x in K562 cells through the activation of PP1 (12). In the present study, we employed the Human Exon 1.0 ST Array, together with the easyExon software, to assess the comprehensive profile of alternatively spliced apoptotic genes in imatinib-treated K562 cells. A total of 175 (175/661, 26.48%) differentially spliced apoptosis-related transcripts were identified, several of which were successfully validated by RT-PCR and sequencing. Our results revealed that imatinib treatment led to aberrant alternative splicing events in K562 cells.

Table IV summarizes the previous studies in the literature that have demonstrated the association of some of these alternatively spliced isoforms with apoptotic events. These genes belong to different cell death protein families, including adaptor proteins, Bcl-2 family, and caspases, in which the splicing mechanism has been shown to modulate both intrinsic (mitochondrial) and extrinsic (death receptor) apoptosis pathways. These two signaling routes to cell death are linked via the Bcl-2 family member Bid, whose alternative splicing was also detected by the exon array in our study. Thus, it is likely that imatinib has wide-ranging effects on apoptosis of K562 cells. In addition, our results suggest the existence of a novel mechanism by which imatinib can induce cell apoptosis, which is the regulation of alternative splicing of a series of apoptotic genes.

Human Exon 1.0 ST Arrays, which are comprised of probesets that cover the entire length of all known transcripts, facilitate the study of gene expression at the exon level (probeset expression) and predict the likelihood of encountering alternative splicing for a given gene. This platform has also enabled detection of specific changes at the gene expression level (transcript cluster expression). Exon array technology has several key advantages over conventional analytical methods, including high-throughput, updated content, probesets designed to span the entire length of a given transcript, and expression analysis at the exon level. In the present study, using RT-PCR and sequencing, two of the three randomly-chosen exons (67%) from the 430 probesets were able to be validated for cell differences in abundance of the respective transcript isoforms. Though the exon array technology is reliable and sensitive enough to detect differential expression at the exon level, there are some important limitations that exist and should be considered when analyzing the exon array results. The sensitivity and specificity of the Affymetrix exon arrays for detecting exon splicing across the whole genome has not yet been defined, at least based on the current published data. Thus, confirmation of the detected exon splicing events requires PCR with primers that flank the exons of interest or RNA deep sequencing to demonstrate consistent differential splicing (21).

In conclusion, using exon arrays, we have discovered that imatinib can produce a wide-ranging effect on the alternative splicing of apoptotic factors in K562 leukemic cells. This information may help to improve the mechanism of imatinib therapy in patients with CML. Even though several of the imatinib-induced alternative splicing events were successfully validated by RT-PCR and sequencing in our study, a more comprehensive validation will be necessary to provide a more accurate estimation of the current findings.

## Figures and Tables

**Table I:** Primers and amplification conditions used in this study.

Gene name	Sequence (5′→3′)	Reaction condition	Splicing isoform	Product size (bp)
*Bcl-x*	F: ATGGCAGCAGTAAAGCAAGCG	32 cycles at 94°C for 30 sec, 55°C for 30 sec, and 72°C for 1 min	Bcl-x_L_	456
	R: TCATTTCCGACTGAAGAGTGA		Bcl-x_S_	267
*RNF34*	F: TGAGTTTCCTGGTAGAGCC	35 cycles at 94°C for 30 sec, 55°C for 30 sec, and 72°C for 1 min	Variant L	173
	R: TTCCCATGACTTCATTCAGC		Variant S	107
*SNCA*	F: GTTGGAGGAGCAGTGGTG	35 cycles at 94°C for 30 sec, 64.5°C for 45 sec, and 72°C for 1 min	Variant L	313
	R: ATGACTGGGCACATTGGA		Variant S	229
*β-actin*	F: CGGGAAATCGTGCGTGAC	35 cycles at 94°C for 30 sec, 55°C for 30 sec, and 72°C for 1 min	-	443
	R: TGGAAGGTGGACAGCGAGG			

**Table II:** Alternative splice probesets that were significantly upregulated in K562 cells in response to imatinib treatment (20 most differentially expressed)^a^.

Gene probe ID	Exon probe ID	Splice index	Gene title	Gene symbol	Accession no.
4007617	4007634	15.12	Pim-2 oncogene	*Pimatinib2*	NM_006875
2638676	2638692	9.74	ELL associated factor 2	*EAF2*	NM_018456
3939545	3939549	9.47	Macrophage migration inhibitory factor (glycosylation-inhibiting factor)	*MIF*	NM_002415
2574752	2574785	8.85	Excision repair cross-complementing rodent repair deficiency, complementation group 3 (xeroderma pigmentosum group B complementing)	*ERCC3*	NM_000122
3818468	3818480	7.39	Tumor necrosis factor (ligand) superfamily, member 9	*TNFSF9*	NM_003811
2939034	2939056	6.77	Serpin peptidase inhibitor, clade B (ovalbumin), member 6	*SERPINB6*	NM_004568
3127703	3127731	6.05	Tumor necrosis factor receptor superfamily, member 10b	*TNFRSF10B*	NM_003842
3736290	3736292	6.00	Baculoviral IAP repeat-containing 5 (survivin)	*BIRC5*	NM_001168
2524743	2524769	5.58	FAST kinase domains 2	*FASTKD2*	NM_014929
3816509	3816515	5.30	Growth arrest and DNA-damage-inducible, β	*GADD45B*	NM_015675
3454662	3454675	5.05	Family with sequence similarity 130, member A1	*FAM130A1*	NM_030809
3868183	3868242	4.80	Interleukin 4 induced 1	*IL4I1*	NM_152899
3790704	3790707	4.62	Phorbol-12-myristate-13-acetate-induced protein 1	*PMAIP1*	NM_021127
3956433	3956483	4.58	CHK2 checkpoint homolog (*S. pombe*)	*CHEK2*	NM_001005735
2940920	2940959	4.50	Eukaryotic translation elongation factor 1 epsilon 1	*EEF1E1*	NM_004280
3886704	3886711	4.50	Serine/threonine kinase 4	*STK4*	NM_006282
3426257	3426261	4.47	Suppressor of cytokine signaling 2	*SOCS2*	NM_003877
2638676	2638690	4.36	ELL associated factor 2	*EAF2*	NM_018456
2940920	2940962	4.29	Eukaryotic translation elongation factor 1 epsilon 1	*EEF1E1*	NM_004280
2589214	2589215	4.27	Protein kinase, interferon-inducible double stranded RNA dependent activator	*PRKRA*	NM_003690

a A more comprehensive list of alternatively spliced apoptotic genes in K562 cells treated with imatinib is available upon request.

**Table III:** Alternative splice probesets that were significantly down-regulated in K562 cells in response to imatinib treatment (20 most differentially expressed).

Gene probe ID	Exon probe ID	Splice index	Gene title	Gene symbol	Accession no.
3742783	3742800	0.21	NLR family, pyrin domain containing 1	*NLRP1*	NM_033004
3481410	3481444	0.21	Tumor necrosis factor receptor superfamily, member 19	*TNFRSF19*	NM_018647
3426257	3426279	0.24	Suppressor of cytokine signaling 2	*SOCS2*	NM_003877
3873160	3873177	0.26	Tribbles homolog 3 (Drosophila)	*TRIB3*	NM_021158
3865301	3865328	0.27	Excision repair cross-complementing rodent repair deficiency, complementation group 2 (xeroderma pigmentosum D)	*ERCC2*	NM_000400
3742783	3742808	0.27	NLR family, pyrin domain containing 1	*NLRP1*	NM_033004
3873160	3873179	0.29	Tribbles homolog 3 (Drosophila)	*TRIB3*	NM_021158
3426257	3426280	0.3	Suppressor of cytokine signaling 2	*SOCS2*	NM_003877
3481410	3481459	0.3	Tumor necrosis factor receptor superfamily, member 19	*TNFRSF19*	NM_018647
3873160	3873178	0.3	Tribbles homolog 3 (Drosophila)	*TRIB3*	NM_021158
3838795	3838806	0.31	Bcl-2-like 12 (proline rich)	*BCL2L12*	NM_138639
3699508	3699557	0.31	Craniofacial development protein 1	*CFDP1*	NM_006324
3924144	3924180	0.31	Collagen, type XVIII, α 1	*COL18A1*	NM_030582
3873160	3873176	0.31	Tribbles homolog 3 (Drosophila)	*TRIB3*	NM_021158
3742783	3742792	0.32	NLR family, pyrin domain containing 1	*NLRP1*	NM_033004
3465409	3465413	0.33	B-cell translocation gene 1, anti-proliferative	*BTG1*	NM_001731
3441849	3441861	0.33	Tumor necrosis factor receptor superfamily, member 1A	*TNFRSF1A*	NM_001065
3441849	3441863	0.33	Tumor necrosis factor receptor superfamily, member 1A	*TNFRSF1A*	NM_001065
3194896	3194952	0.33	TNF receptor-associated factor 2	*TRAF2*	NM_021138
2487412	2487447	0.34	Annexin A4	*ANXA4*	NM_001153

**Table IV:** Apoptotic genes previously shown to be regulated by alternative splicing.

Gene symbol (ref.)	Gene title	Accession no.	Gene probe ID	Exon probe ID	Splice index
Adaptor proteins and regulators					
*TRAF2* (22)	TNF receptor-associated factor 2	NM_021138	3194896	3194919	0.50
				3194950	0.50
				3194944	0.48
				3194952	0.33
*Apaf-1* (23)	Apoptotic peptidase activating factor 1	NM_181868	3427876	3427877	3.68
				3427936	3.00
				3427937	2.46
				3427881	2.26
				3427889	2.08
Bcl-2 family					
*Bcl-x* (24)	Bcl-2-like 1	NM_001191	3902489	3902523	0.4
*Bak* (25)	Bcl-2-antagonist/killer 1	NM_001188	2950753	2950768	2.14
*Bax* (26)	Bcl2-associated X protein	NM_004324	3838067	3838084	0.48
*Bid* (18)	BH3 interacting domain death agonist	NM_197966	3951927	3951950	3.91
				3951929	3.64
				3951928	2.63
				3951930	2.15
*Bmf* (17)	Bcl-2 modifying factor	NM_001003940	3619229	3619242	3.59
Caspases and caspase-like proteins					
*Caspase-3* (19)	Caspase 3, apoptosis-related cysteine peptidase	NM_004346	2796484	2796499	2.00
				2796505	2.00
*Caspase-10* (27)	Caspase 10, apoptosis-related cysteine peptidase	NM_032977	2522693	2522719	2.33
				2522707	2.07
				2522715	2.00
